# Industry 4.0 Technologies Applied to Inland Waterway Transport: Systematic Literature Review

**DOI:** 10.3390/s22103708

**Published:** 2022-05-12

**Authors:** Juan Felipe Restrepo-Arias, John William Branch-Bedoya, Julian Andres Zapata-Cortes, Edwin Giovanny Paipa-Sanabria, Miguel Andres Garnica-López

**Affiliations:** 1 Facultad de Minas, Universidad Nacional de Colombia, Medellín 050041, Colombia; jwbranch@unal.edu.co; 2 Fundacion Universitaria CEIPA, Sabaneta 055450, Colombia; julian.zapata@ceipa.edu.co; 3 Cotecmar, Cartagena 130001, Colombia; epaipa@cotecmar.com (E.G.P.-S.); miguel.garnica@armada.mil.co (M.A.G.-L.)

**Keywords:** Industry 4.0, waterway inland transport, systematic literature review, artificial intelligence

## Abstract

The focus of this article is inland waterway transport. Different problems in this domain have been studied due to the increase in waterway traffic globally. Industry 4.0 technologies have become an alternative for the possible solution of these problems. For this reason, this paper aims to answer the following research questions: (1) What are the main problems in transporting cargo by inland waterway? (2) What technological strategies are being studied to solve these problems? (3) What technologies from Industry 4.0 are used within the technological strategies to solve the exposed problems? This study adopts a Systematic Literature Review (SLR) approach. For this work, were recovered 645 articles, 88 of which were eligible, from which we could identify five domains corresponding to (1) traffic monitoring, (2) smart navigation, (3) emission reduction, (4) analytics with big data, and (5) cybersecurity. The strategies currently being considered combine navigation technologies, such as AIS (Automatic Identification System), which offers a large amount of data, with Industry 4.0 tools and mainly machine learning techniques, to take advantage of data collected over a long time. This study is, to our knowledge, one of the first to show how Industry 4.0 technologies are currently being used to tackle inland waterway transport problems and current application trends in the scientific community, which is a first step for the development of future studies and more advanced solutions.

## 1. Introduction

We are currently experiencing a change in the global production paradigm known as the Fourth Industrial Revolution, in which different industrial sectors and connectivity are integrated through the Internet of Things (IoT) and cyber–physical systems to make Industry 4.0 reality [1]. The leading technologies associated with Industry 4.0 are: 3D printing [2], robotics, [3], Unmanned Aerial Vehicles (UAVs) [4,5], Internet of Things (IoT) [6], blockchain [7], and artificial intelligence [8].

Although the applications of Industry 4.0 technologies have had a more specialized focus on the manufacturing industry [8]. According to [9], logistics service companies have begun to study problem-solving and to improve their services, relying on these technologies, and the main applications in the logistics sector can have four main objectives:

- Greater speed: delivery services performed by autonomous vehicles or delivery robots.
- Greater reliability: storage and retrieval systems using robots.
- Lower operating cost: inventory monitoring and replenishment systems using intelligent sensors.
- Better efficiency: blockchain-enabled container shipping. Because ocean freight operations involve many organizations and paperwork, lengthy and uncertain delays are commonly seen because many processes are done manually.

Inland waterway transport is an essential element of integrated transport systems. Compared to other transport modes, inland waterway transport is characterized by high transport volume, low energy consumption, and low environmental impact [10].

Despite the numerous advantages of river navigation, it is the least developed compared to road and rail transport due to the combination of several factors, mainly in developing countries. General factors such as infrastructure deficiencies, low investment, lack of strict regulations, and lack of coordination between national and sub-regional institutions challenge the proper functioning of inland waterway transport [11].

The solution to these and other problems associated with river navigation constitutes research challenges, which can be addressed from Industry 4.0. The main objective of this work was to do a systematic literature review to detect the problems that are being solved with the help of the technologies associated with Industry 4.0. Therefore, the following research questions are considered for the present SLR:

- What are the main problems in transporting cargo by inland waterways?
- What technological strategies are being studied to solve these problems?
- What technologies from Industry 4.0 are used within the technological strategies to solve the exposed problems?

This paper is organized as follows: in Section 2, the methodology is explained. Section 3 presents the answers to the research questions. In Section 4, the SLR limitations are presented, and finally, Section 5 presents the conclusions and future work.

## 2. Methodology

### 2.1. Selection Criteria

To guarantee the quality of the articles, only those that passed the following selection criteria were considered in the review process:

- Papers published in peer-reviewed conferences, peer-reviewed journals.
- Documents published in English and Spanish.
- Documents that answered any of the three research questions.
- Documents published between 2016 and 2021 (both years inclusive).

After searching the libraries and analyzing the titles, it was removed if the main topic of a given article was irrelevant or outside the scope of this study. Additional selection criteria were then applied to reduce the number of articles found on the search and obtain several high-quality sources that could be used to answer the research questions. This additional criterion was based on two specific inclusion sub-criteria, which were defined in a two-stage process:

Inclusion criteria based on abstracts: in this phase, the articles found in the search stage based on the information provided in their abstracts were discarded. The articles that met this inclusion criterion were kept for further processing, that is, works that discussed problems and Industry 4.0 technologies applied to inland waterway transport. Papers with little relevant information in their abstracts were temporarily kept on the list and processed in the next stage.

Inclusion criteria based on complete reading: in this phase, articles that did not answer the research questions were eliminated, that is, although the key search terms that are shown in Table 1 appeared in the articles, if they did not indicate to answer the research questions, they were discarded.

### 2.2. Information Sources

An internet search was performed using some of the most important search engines for scientific articles and academic information. The obtained results were collected manually to select the best sources of information to answer the aforementioned research questions. After analyzing the results, the digital libraries described in Table 2 were chosen based on their scientific and technical content and their relationship with the engineering and technologies associated with the objective of this work.

These information sources allow the use of search algorithms composed of logical operators that are useful in extracting the desired information to perform the systematic review.

### 2.3. Search Strategy

The next step was to define key search terms and a consistent procedure to search for scientific and technical documentation in the chosen digital libraries. First, a set of keywords associated with the research questions were selected, with which, three groups of words were created, which are shown in Table 1. Each group contained consolidated expressions with synonyms or terms with related meanings.

Group 1 included terms associated with river navigation, while group 2 contained a set of general terms related to technologies that have been commonly used in solving river navigation problems. Finally, group 3 included terms associated with Industry 4.0 technologies. Logical operators supported by the advanced search of digital libraries were used to build search strings based on the three research questions, combining terms from groups 1 and 2 in the first algorithm and groups 1 and 3 in the second algorithm search.

The general structure of the search queries that were applied to the information sources is shown in Table 3.

### 2.4. Selection and Collection Process

The search process was conducted using the words from the groups in Table 1 to define the algorithm search queries given in Table 3 used in the digital libraries. The articles search process was limited to title, abstract, and keywords in the databases. This search process was carried out by one researcher.

After that, we reviewed each article that passed the initial eligibility criteria with a detailed lecture to detect the most important technologies used in these solutions. This was done by pairs of researchers and a consensus was reached by discussion. In the process, we detected articles that did not use any technology related to Industry 4.0 and, therefore, were excluded. This process left us 88 studies. A total of 100% of the included articles were retrieved only from Scopus since the articles selected in the early stages of Web of Science did not meet the selection criteria.

**Figure 1:**
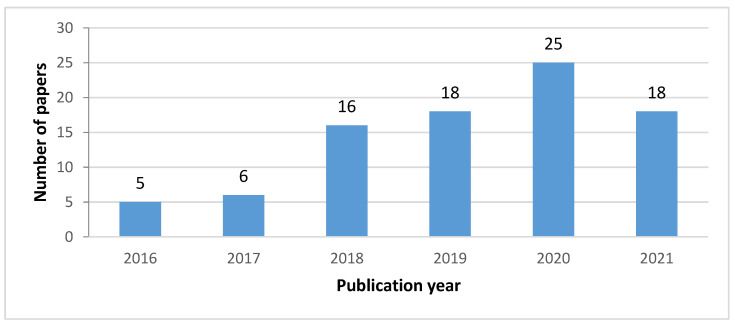
Distribution of the extracted papers by publication year.

The papers’ distribution by years can be seen in Figure 1, where the growing trend in recent years can be seen.

**Figure 2:**
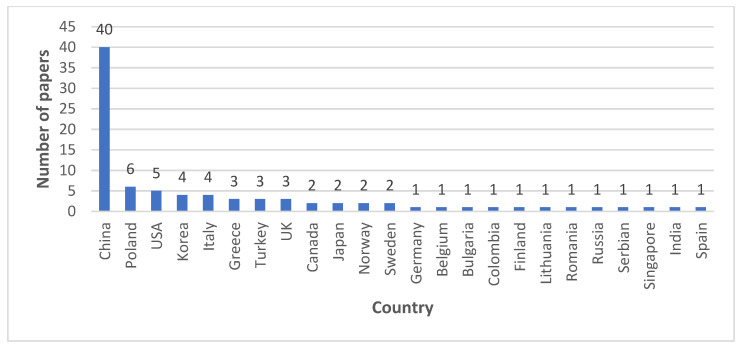
Country distribution of the selected articles.

In Figure 2, it can be seen that the most significant number of selected articles were developed in China, with a high difference over other countries.

The protocol utilized in this systematic review [12] offers certain advantages. First, it speeds up the search for domains that have limited available literature, allowing for rapid identification of recognized authors and research centers within that specific domain. It also allows one to quickly identify technologies or groups of technologies used for further, more detailed analysis.

However, the protocol used may have weaknesses in the evaluation of the quality of the papers, since a rigorous analysis of the evaluator pairs was not carried out, it was only based on the reliability of the scientific databases. Another weakness may be the search strategy in which keywords may have some bias due to particular search interests that are considered.

## 3. Results

**Figure 3:**
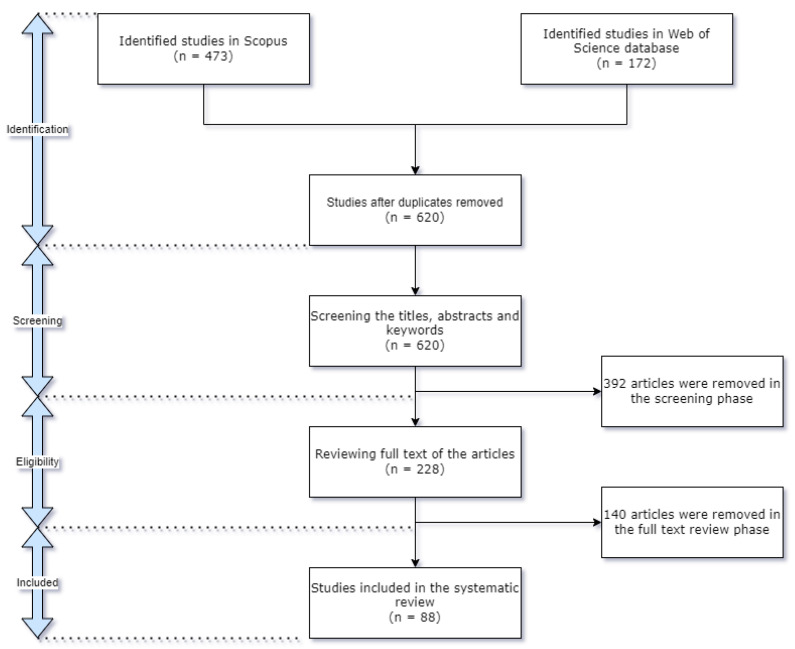
Summary review protocol.

This phase presents the results of the SLR in order to answer the aforementioned research questions based on the extracted information from the main studies selected. Initially, 645 studies were screened from the chosen electronic databases. These were distributed as follows: 176 Scopus, groups 1 and 2; 297 Scopus, groups 1 and 3; 162 Web of Science, groups 1 and 2; and 10 Web of Science, groups 1 and 3. In the first place, duplicates were excluded, that is, the studies available in the two databases, eliminating 25 copies, and then the articles that did not meet the research questions and the context of the search were extracted, which totaled 392 articles. Then, the summary reading criterion was first applied to the remaining 228 studies, which resulted in 88 articles, which were given a complete reading (Figure 3).

### 3.1. Answer to the First Research Question

What are the main problems in transporting cargo by waterways? In order to identify the main research problems addressed in the different studies around inland waterway transport, the papers that were finally reviewed were grouped into five domains corresponding to: (1) traffic monitoring, (2) smart navigation, (3) emission reduction, (4) analytics with big data, and (5) cybersecurity.

**Figure 4:**
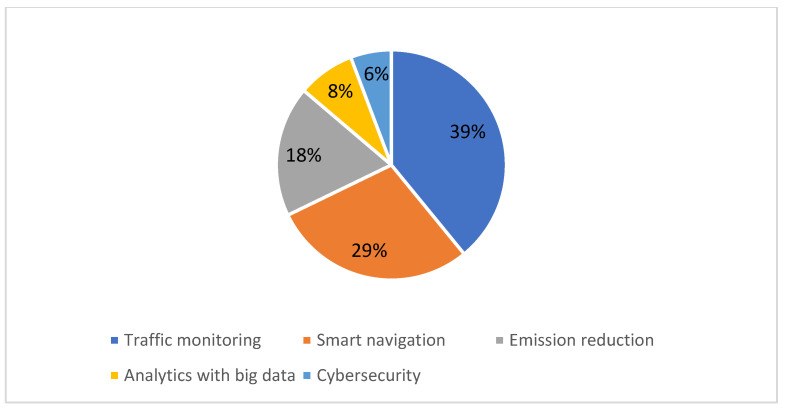
Distribution of the articles selected by application domain.

These domains were named in this way to group the different works retrieved in the SLR, which have these five characteristics in common. It is important to mention that the name of the fourth domain, “analytics with big data”, refers to the analytical work carried out with data that was traditionally analyzed with other tools in the inland waterway transport domain. It is important to note too, that although the focus of this study is on river navigation, some works refers to maritime transport. However, those considered relevant to the study were included from this domain because they meet two criteria: (1) they refer to high-traffic areas such as the entrance to ports, which are even located at the mouths of large rivers, and (2) they refer to technologies that can be applied to solving river navigation problems. The results are summarized in Table 4 and illustrated in Figure 4. From this figure, it can be seen that most of the selected studies focused on the study of traffic monitoring (39%), followed by smart navigation (29%), emission reduction (18%), analytics with big data (8%), and cybersecurity (6%).

#### 3.1.1. Traffic Monitoring

**Figure 5:**
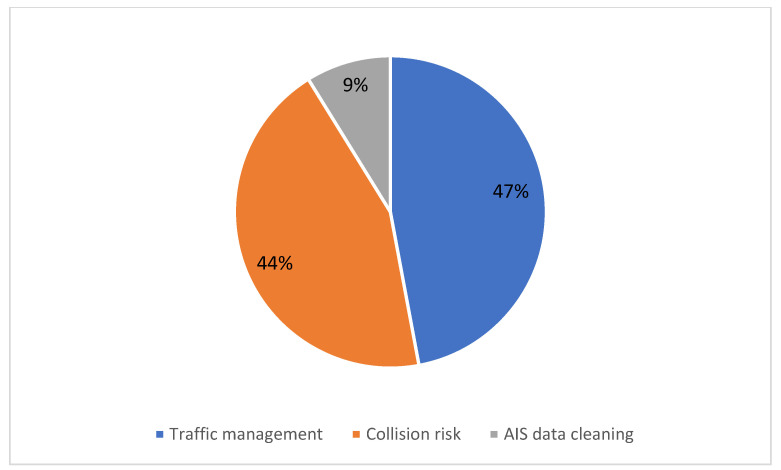
Subdomains in traffic monitoring.

The grouped and selected articles in the traffic monitoring domain show a greater interest in solving problems associated with the high volume of vessels that currently navigate the world’s seas and rivers. Specifically, the following three subdomains were identified: (1) traffic management (47%), (2) the risk associated with collisions between vessels (44%), and (3) correction and cleaning of data mainly from AIS (Automatic Identification System) (9%) (Figure 5). Some characteristics addressed by the researchers are described below:

- **Traffic management:** This subdomain focuses mainly on route recognition, congestion, and prediction of arrival times at the destination [14,15,17,19]. One of the goals is to improve operational efficiency of container terminals. AIS data are used as a research basis to predict the arrival time of ships and reduce uncertainty [13,24,26] to provide support for the construction of intelligent ports [19]. The specific identification of vessels is also one of the areas of most significant interest. Being able to identify vessels with images from different angles [21,28], as well as recognizing their identification numbers [41], are key factors to be able to keep statistics on port arrivals and traffic control. Researchers have been experimenting with deep learning methods, specifically those developed with networks that allow real-time detection, such as YoloV3, to solve this problem [21]. Recognizing flow patterns in waterways has also been the subject of research to estimate the hours of greatest flow and regulate traffic in critical areas [25]. The technologies used to capture images in this subdomain are diverse, such as hyperspectral cameras mounted on crewed planes [20], satellite images [22,47], and video and photography cameras [27,28] which are the most commonly used image sources. The primary data source for this subdomain is the AIS system, [19,26,32,101]. However, all studies about AIS refer to the pre-processing for the data cleaning that must be done.
- **Collision risk detection:** This subdomain is also one of the most active research areas. In the search results, studies that focus on waterways are included, and those carried out on traffic around seaports, since many of them are located at the mouths of large rivers [32,33,34]. There are high risks of collision in these contexts, and different methods are being explored to detect the risk promptly. One of the problems addressed in this subdomain is the complexity of managing all the data through ECDIS (Electronic Chart Display and Information Systems), which can be saturated with data and do not allow refined information to be obtained to detect possible risks quickly by a part of the pilots. In addition, the costs of changing the navigation route can be very expensive when a possibility of collision is faced [33,35].
  The technologies that have been tested in this subdomain are mainly neural networks, based on images captured through cameras located along waterways or at strategic port entry and exit sites [42]. The goal is to avoid collisions in high-traffic areas. For example, in [33], ship features are extracted layer by layer through the DarkNet53 network, and multi-scale image pyramid features are formed to detect ships of different sizes. Specifically, in this research, four typical port navigation scenes have been selected: (1) small traffic flow, (2) boat navigation with fog, (3) large traffic flow, and (4) small image scale. Experiments show that yolov3-based ship detection has high accuracy in the face of complex sea and river navigation conditions and can cope with the detection requirements of different scenes [42].
- **AIS data cleaning:** The third subdomain in traffic monitoring comprises the research papers that focus on one of the main problems reported in the other subdomains: data cleaning and correction. The most used data come from AIS systems. However, these data about the ship trajectory inevitably bring noise or missing data that can interfere with accurate information [36]. Therefore, some of the most used methods have to do with interpolation techniques to reconstruct sections of trajectories that have been lost. Enhanced kinematic reconstruction, for example, includes four steps: (1) data pre-processing; (2) time interval distribution analysis; (3) abnormal data detection and removal, and (4) kinematic interpolation that takes the kinematic feature of ships (i.e., speed and acceleration) [36]. One of the most used methods for solving this problem is spatial clustering, based on the density of applications with noise or density-based spatial clustering of applications with noise (DBSCAN) [37]. DBSCAN can be applied to historical data or real-time Automatic Identification System (AIS), so that ship routes can be modeled and trajectory anomalies can be detected. This technique is combined with others based on neural networks, which work specifically with sequential data. For example, in [43], the density-based clustering method is introduced in the first phase to recognize the undesirable outliers in terms of DBSCAN automatically, and in the second phase, a bidirectional supervised learning technique based on DBSCAN is proposed. Furthermore, long- and short-term memory (BLSTM) restores time-stamped points degraded by random outliers in ship tracks.

#### 3.1.2. Smart Navigation

**Figure 6:**
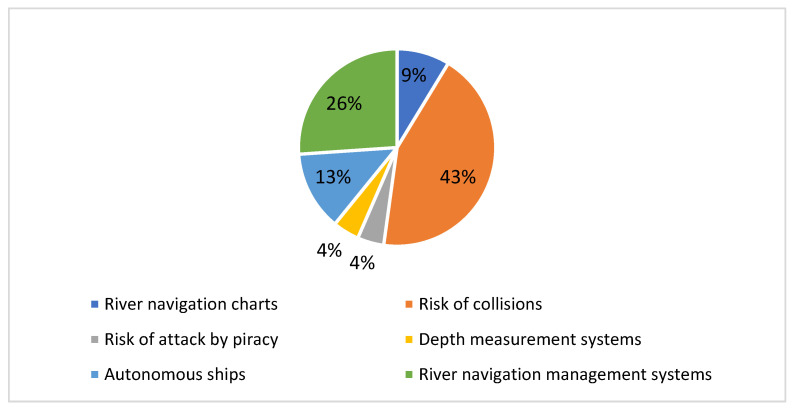
Smart navigation subdomains.

The articles selected and grouped in the smart navigation domain are varied. There seem to be diverse interests in solving problems associated with different themes: (43%) risk of collisions, (26%) river navigation management systems, (13%) autonomous ships, (9%) river navigation charts, (4%) depth measurement systems, and (4%) risk of attack by piracy (Figure 6). Some of the characteristics of this subdomain are set out below:

- **Collision risk:** Unlike the first domain, where there is also a majority interest in the problems associated with the risk of collisions, in the smart navigation domain, the problem is addressed from the perspective of data collected on the vessel itself, and in that the technologies are associated with internal sensors, also in order to avoid obstacles [49,50,51,52,53,54,55,56,57,58,59,61]. For example, authors in [60] proposed a method based on the measurement of approach speeds to detect collision candidates. Collision candidates are detected based on a perspective that considers a ship encounter as a process instead of analyzing traffic data at particular time intervals. Case studies on unique vessel traffic encounters in high traffic waterway environments are conducted and presented in this paper.

- **River navigation management systems:** One of the subdomains that have been considered important in smart navigation refers to the management of waterways from land [62]. Among the possible classifications of the issues that can be addressed with this perspective are waterways monitoring, capturing and storing waterway data, maintenance management of river corridors, and public waterway information services [63,64,65,66]. One of the main pieces of technology in inland waterway transport, the River Information Services (RIS), is used similarly like a source of data, in combination with some technologies of Industry 4.0 in this subdomain [65,70]. However, research studies that use RIS with Industry 4.0 technologies were scarce, at least in the databases searched, as only three were retrieved, which is of note because RIS is a well-known technology in the river transport sector.
- **Autonomous ships:** This subdomain addresses the problems associated with autonomous navigation in its entirety, where all the associated technologies are combined [59,61]. The recovered studies in this area are still scarce, and all are pilot projects in which there are still many tests to overcome. According to [102], there are four levels of autonomous navigation: (1) conventional ships with an automated decision support system, for example, a collision-avoidance system, (2) periodically autonomous ships, that is, autonomous functions are activated at night, and in good weather, (3) fully autonomous ships with crew facilities to take ships in or out of ports, and (4) fully autonomous ships without crew facilities on board. According to the same author, a coastal control center managed by people from land will always be necessary for alternatives two and three. Some studies propose autonomous navigation supported by aerial devices to reduce the risk of collision. For example, the authors in [55] proposed in 2018 an autonomous navigation system supported by unmanned aerial vehicles (UAVs) to detect objects close to the boat.
- **River navigation charts:** Knowledge about the network of rivers and canals in a territory is one of the problems addressed in this subdomain. It is considered essential to have a priori information on how the waterways are distributed and to keep this information updated [54]. In this sense, the authors in [65] proposed, for example, solving this problem using automated hydrographic survey systems, which allow the rapid conduct of inland surveys with reasonably low operating costs. According to authors in [65], this type of information will help establish smart navigation and lead to broader use of the region’s inland waterways by cargo and passenger ships in the future.
- **Depth measurement systems:** Real-time detection of waterway depth is crucial for smart navigation in these types of corridors. This problem is associated with waterways that can represent risks of accidents, and although studies are scarce, they require the attention of researchers. In [50] was proposed a model that combines a decision tree and a neural network, trained and tested with data from a AIS (Automatic Information System) and Global Mapper from the ports of Nantong and Fangcheng on the southeast and southwest coasts of China.
- **Risk of attack by piracy:** The risks of attack by piracy are widespread in some maritime areas. For a decade, the phenomenon of piracy has been studied, mainly in the maritime sector [103]. However, waterways are not exempt from running these same risks, especially in countries with growing security problems. To counteract this threat, some researchers have proposed models that combine information from onboard sensors and cameras with intelligence from external sources for early detection of hacking threats, which have shown promising results. However, it lacks real-time updates for the context of the situation [48].

#### 3.1.3. Emission Reduction

Emissions reduction is one of the most critical domains in this classification of recovered studies. Therefore, the maritime and river sectors must make a great initial effort to reconvert technologies to minimize greenhouse gas (GHG) emissions [73,74]. For example, heavy-duty diesel engines are widely used in large ships, and are used primarily in maritime transport, and are the main contributors to greenhouse gas emissions and other pollutants, such as CO_2_, NO_X_, SO_X_, and particulate matter (PM) [104]. On the other hand, in smaller vessels, with a power range between 200 and 1000 kW, which are the most abundant in river navigation, the vast majority of engines use gasoline engines [79]. This sector faces a challenge in advancing towards cleaner technologies that use fewer fossil fuels.

**Figure 7:**
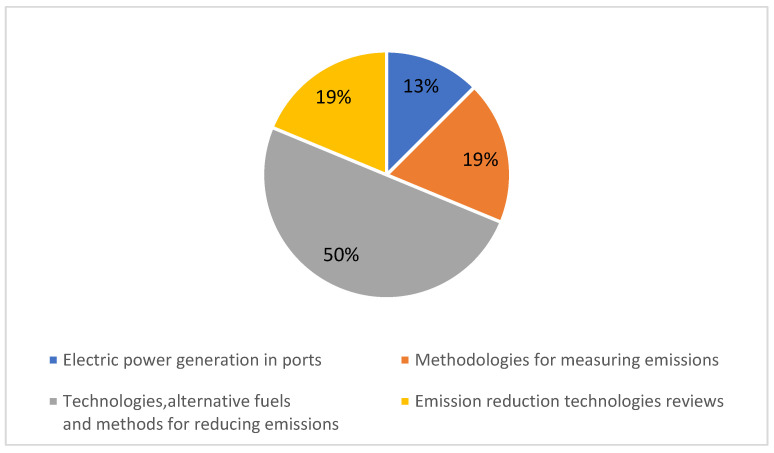
Emission reduction subdomains.

The papers selected and grouped in the emissions reduction domain were classified into four subdomains: (50%) technologies, alternative fuels, and methods to reduce emissions; (19%) methodologies to measure emissions; (19%) literature reviews on technologies to reduce emissions; and (13%) power generation in ports (Figure 7).

- **Technologies, alternative fuels, and methods for reducing emissions:** There are different research approaches in this subdomain:
  ○ Measuring and recommending approach speeds to ports [86].
  ○ Designing the shape of vessels to make them more efficient and friendly to protected environments [81].
  ○ Use of heat losses to produce electrical energy [82].
  ○ Propeller selection methods for electric motors [85].
  ○ Use of alternative fuels such as methanol, ammonia, and hydrogen [88].
  ○ Reaching emerging technologies such as ships propelled with solar energy [87].
  In this same domain, according to [83], three of the technologies in which greater interest has been placed for the reduction of emissions in river navigation are (1) dual LNG engines (Liquefied Natural Gas), which are engines that use natural gas and diesel fuel bunker; (2) Hybrid propulsion systems, in which thermal engines are combined with electric engines. The advantages of a hybrid engine are associated with integrating different energy sources, which can be used because they run on engines with different high-efficiency regimes, which yields a much higher overall efficiency than conventional engines; (3) Microturbines represent an innovative way of producing electricity at moderate costs and with almost zero emissions. In hybrid solutions, the microturbines operate with batteries stored on board to provide the necessary electrical energy.
- **Methodologies to measure emissions:** The precise measurement of greenhouse gas emissions is also an important field of research, mainly due to the importance of measuring the impact that new technologies can generate in reducing the generation of harmful gases. The authors of [76] proposed a method to address this technical need, in which they developed a novel approach that can incorporate the strategic implementation of fuel options and quantify their suitability to comply with future environmental pollution legislation. The core algorithm of this approach is based on large sample size probabilistic simulations of ship movement in the designated port area, using a Bayesian ship traffic generator from existing actual activity data [76]. In addition, the measurement of the impact of fuels such as hydrogen is also an active field of research, and some authors propose measurement methods, which include comparisons with technologies such as electric power and their stages of development and legislation [75].
- **Literature reviews on emission reduction technologies:** It is essential to highlight that three literature reviews were included in this domain that makes a detailed classification and analysis of the currently studied technologies to reduce emissions in the maritime and river sector [84,85,86]. Although it is not common to include review studies in a first-order systematic review like this one, we considered including them due to the scarcity of reviews in this sense that have been done to date, and which, due to their rigor and timeliness, can serve as a guide for future studies. To alleviate the impact of global shipping on the environment, the international maritime organization (IMO) established stricter emissions regulations from tier I to tier III in order to reduce ship emissions [78]. The authors in [78] conducted an exhaustive literature review based on the different levels established by the IMO. Based on a significant number of related pieces of literature, they concluded that using alternative fuels to reduce polluting emissions is gradually becoming generalized. In particular, liquid natural gas (LNG) is considered one of the most promising alternative fuels due to its economic and environment-friendly characteristics. This review aims to summarize the different emission reduction technologies of diesel engines through three reduction pathways: (1) fuel optimization, (2) pre-combustion control, and (3) exhaust gas post-treatment escape. In addition, the use of LNG in marine diesel engines was evaluated. In [80], the authors identify promising technologies and practices applicable to all-electric shipboard power systems and reveal the energy efficiency of all-electric ships in different applications. This document suggests that the proposed strategies should eventually be combined with alternative technology and operations-based measures, as implemented in conventionally powered vessels, to realize the full potential of an energy-efficient operation. Finally, in [79], the leading solutions currently being developed or adopted for low- and medium-speed diesel engines have been reviewed from a qualitative and sometimes quantitative point of view. Nevertheless, compared to the less current literature, focusing more on their potential concerning the possible use of waste heat recovery systems, such as, in particular, Steam Rankine Cycles and Organic Rankine Cycles (ORC).
- **Generation of electrical energy in ports:** The generation of electrical energy with different alternatives in the port area is an area of research that has been gaining strength to supply energy to new vessels with AES electric motors (all-electric ships). In this sense, in [73], the authors study micro-grids in ports, which are based on relatively mature technologies and can bring enormous economic and environmental benefits. However, they concluded that there are still some gaps in knowledge to solve before these technologies can be used in maritime applications. With this perspective, this overview study emphasizes the characteristics of the seaport micro-grid and AES. Then, various emerging technical challenges and future research prospects were raised after a comprehensive study of the literature. The authors in [74] provided a comprehensive review of the technical aspects, practices, existing standards, and critical challenges in designing and modeling a port network for a shore-to-ship power supply. This paper presents current and future solutions that discuss shore-to-ship power technology and considers the voltage, frequency, power, and other technical requirements of onboard and shore-based vessels. In addition, this study contributes to designing suitable models for smart grids in the port area that can facilitate both shore power supply and battery charging for future hybrid and electric ships.

#### 3.1.4. Analytics with Big Data

The fourth domain detected in the literature review was due to the opportunities offered by the speed, volume, and variety of the data that has been collected from different sources in the maritime and river sector, such as from Automatic Identification System (AIS), RADAR, LIDAR, or VDES [89,90,91,92,93,94,95]; identification and long-range tracking; radar tracking; remote sensing; Internet of Things; among others. Currently, this system generates opportunities to calculate new metrics, which serve to measure efficiency in transport by fluvial and maritime routes, and which in turn help technicians and researchers in the area of logistics [93]. Furthermore, simulation processes of the river and maritime traffic, which have been the focus of study since the 1960s and 1970s, have benefited from this large amount and variety of data since new methodologies have been proposed to support these simulation processes of the traffic that now have more and better data [92,95]. Based on this increasingly available information on ship traffic at sea. In general, data-driven knowledge discovery has recently demonstrated its value in fields that go beyond the original maritime security functions of such data [90]. The authors in [90] included but are not limited to fisheries management, maritime spatial planning, the ship emissions grid, at-sea mapping activities, offshore shelf risk assessment, and trade indicators. In conclusion, all the data that has been extracted from the different sources will be a crucial element in the formulation of regulations and as an essential source of necessary research in the maritime and river sectors, which will probably allow us to have a better understanding of these sectors. However, conventional analysis techniques do not have the robustness necessary to process this volume of data, and the use of techniques associated with Big Data is becoming more noticeable.

#### 3.1.5. Cybersecurity

According to the authors in [96]:

“…2017 saw a proliferation of cyber-attacks, showing that the cyber threat landscape is complex and constantly changing. To answer this, marine and offshore organizations need to take a more strategic approach to protecting their critical assets and business drivers. They need to build secure and scalable security postures by deploying comprehensive, multi-layered defenses that are risk-based and threat intelligence-led. They need to cover not just technology but people and processes as well to ensure that technologies are properly configured so as to step up to the increasingly complex challenges that face them”.

In this domain, some initiatives aim to strengthen the framework of the guidelines and create cybersecurity systems since there are still significant weaknesses in this aspect [96,97,98,99,100]. According to [98], the current maritime cybersecurity guidelines have two main flaws: (1) they do not provide a holistic set of recommendations to key stakeholders in the shipping system and (2) the current guidelines are not sufficiently grounded in research. This paper also proposes a scheme to enable stakeholders to develop comprehensive and more effective cybersecurity guidelines for the shipping industry [98].

The cybersecurity of ports has begun to be a focus of interest on the part of researchers. For example, the authors in [99] proposed to apply an integrated cyber risk assessment method for a container port with a cyber-physical perspective by analyzing four exemplary cyberattack scenarios. The authors apply a risk assessment methodology using an integrated cybersecurity management approach for each cyberattack scenario. In addition, for the specified cyber threats, the risks have been evaluated as unacceptable, and in the end, they briefly present some strategies for their mitigation. In general, it was detected in the literature that cybersecurity in maritime and river navigation is still an area of study that is just beginning, and in which it is necessary to establish robust guidelines that allow the different interested entities to take advantage of existing tools, and the possible developments that are made around this topic. In [97], the authors reviewed several approaches to maritime cybersecurity, outlined available resources, and discussed in their paper how advanced methods, including optical communications and quantum encryption, will improve maritime cybersecurity.

### 3.2. Answer to the Second Research Question

What technological strategies are being studied to solve these problems? Technological strategies refer in this study to the combination of technologies and methods, which appear in the reviewed literature as possible solutions to the problems identified in the six domains.

#### 3.2.1. Strategies in Traffic Monitoring

Two strategies appear. The first is the use of data from an AIS (Automatic Identification System), in which some data cleaning and correction techniques are proposed, with statistical tools, and then mainly Machine Learning techniques are applied. Most of the work employing these strategies uses deep neural networks. The most used of the classic Machine Learning techniques is the DBSCAN algorithm for clustering by density. The second strategy is based on detection with images from different sources: RGB cameras, hyperspectral satellite images, thermal images, and video. Increasingly accessible data sets such as the HRSC2016 dataset or the SSD2020 dataset are also being used. In this second strategy, more and more Deep Learning techniques are being implemented, although classical computer vision techniques are still used in a few works. These two strategies and the associated technologies can be seen in Table 5.

#### 3.2.2. Strategies in Smart Navigation

In this domain, the main objective is to achieve safe navigation, automatically detecting the presence of natural obstacles and other vessels to avoid collisions. Two strategies were identified to achieve this goal. The first has to do with the automatic identification of collision risks, where a combination of different technologies is observed and mainly used inside ships. The second strategy focuses on detecting risks from the ports to provide support to the ships, as seen in Table 6.

#### 3.2.3. Strategies in Emission Reduction

In this domain, there is still not enough evidence of the use of Industry 4.0 technologies in the search for solutions. The strategies are based on “hard technologies”, such as electrical power sources or alternative fuels. However, it is important to mention them because the evidence suggests that solutions based on Industry 4.0 technologies could be explored in this domain. Two strategies were identified in this domain. The first one aims to make efficient use of ship designs, use fossil and alternative fuels with lower emissions, and combine different energy sources. The second one aims to altogether dispense with fuels that generate emissions, emphasizing the use of electric and solar energy, as seen in Table 7.

For the two final domains, analytics with big data and cybersecurity, the studies focused mainly on applying technologies that are transversal to any domain and do not constitute an independent field of study, so no relevant strategies of the domain were identified from the inland waterway navigation sector. However, the application of the methods are common to all domains.

### 3.3. Answer to the Third Research Question

What technologies from Industry 4.0 are used within the technological strategies to solve the exposed problems? Analysis:

- **Neural networks for trajectory prediction:** Technologies from Industry 4.0 used in the strategies mentioned above are mainly from the field of Machine Learning, focused on the field of Deep Learning (deep neural networks). There is a particular emphasis on neural networks specialized in sequential data, such as LSTM or GRU, which are applied to data from time series, such as those obtained from AIS systems.
- **Neural networks for vessel detection:** Artificial vision in the navigation domain has been using networks similar to those observed in other domains. Since their applications are extensive, networks such as Yolo, DarkNet, Autoencoders, and SSD networks, among others, are currently the state-of-the-art in this domain as well.
- **Grouping algorithm—DBSCAN:** In the grouping of data, the predominance in using the DBSCAN method is noted. The main objective of this algorithm is to group by the density of points. This is observed in data obtained from AIS, in which high density is one of its main characteristics.
- **Internet of Things:** The Internet of Things has greatly strengthened data capture, and therefore, AIS systems have benefited from its growing use. Most of the papers reviewed use data from AIS systems, with some IoT elements. This technology has one of the greatest potentials presented in inland waterway transport. The evidence found suggests that it is one of the tools that are most used to collect data, and is gaining more and more interest from researchers in inland waterway transport domains.
- **Big Data:** Big Data technologies, such as non-relational databases, the use of the MAP REDUCE algorithm, and the HADOOP and SPARK frameworks have been increasingly used in managing data coming from AIS systems. These tools are the same as those used in other domains, and their implementation has also been taking place in the fluvial and maritime navigation domains. The fundamental challenge found in the reviewed works is the cleaning and treatment of noise (outliers) and missing data, for which there is still no strategy that stands out in this domain. This seems to be a fundamental problem of the AIS system, for which research is just beginning to provide some answers.

## 4. Limitations of this Study

The SLR based on the protocol used may have weaknesses in the evaluation of quality of the papers. Since a rigorous analysis of the evaluator pairs is not carried out, it is only based on the reliability of the scientific databases. Another weakness may be the search strategy in which keywords may have some bias due to particular search interests that are considered. There is also no detailed analysis of each of the recovered works, which could ignore some failures in the obtained results from the articles reviewed. However, the protocol used speeds up the search in contexts in which there is not very abundant literature, such as the one studied, so recognized authors or research groups in the domain of interest can be quickly identified. It also allows to quickly identify technologies or groups of technologies used for further more detailed analysis.

Another limitation regards the scientific databases. The selection of only two, Scopus and Web of Science, was because a previous search showed us better results in the number of papers recovered compared to others of similar importance. However, there are some important databases that may have articles of interest for a similar study.

An important disadvantage of this study was that technological advances that some companies or important organizations did not publish in scientific articles were left out. For example, no evidence was found of the work from the European Inland Waterway Transport Platform (EU-IWT), or the Innovation & Greening Committee, which were launched in 2019 in order to promote relevant aspects of river transport and specifically in the development of new technologies [105].

Finally, the evidence suggests there is not abundant literature about the applications of Industry 4.0 technologies in inland waterway transport, at least in the databases consulted.

## 5. Conclusions and Future Works

In this paper, we present a systematic literature review on the studies related to Industry 4.0 technologies applied in inland waterway transport. With this, we aimed to recognize the most important problems in river navigation and how these have been solved with Industry 4.0 technologies. We started our SLR by establishing three research questions: (1) What are the main problems in transporting cargo by inland waterways? (2) What technological strategies are being studied to solve these problems? (3) What technologies from Industry 4.0 are used within the technological strategies to solve the exposed problems. To answer these questions, we reviewed the last six years of studies oriented to solve inland waterway transport problems using Industry 4.0 technologies by proposing multiple query algorithms that were used in two digital libraries named Scopus and Web of Science, from which we extracted the studies. Then, we filtered through them using inclusion and exclusion criteria to select the relevant studies to answer our research questions.

This paper shows findings in the literature reviewed, which suggest that the main problems in inland waterway transport are associated with increased traffic volume, which increases the risk of collision accidents. Therefore, the main studied solution was traffic monitoring, followed by smart navigation, which according to the literature review, is still in its infancy. Third, emission reduction, and finally, analytics with big data and cybersecurity.

Additionally, we identified primarily four Industry 4.0 technologies that make contributions to tackle the existing problems in inland waterway transport: Artificial Neural Networks (ANN), Internet of Things (IoT), Big Data, and Grouping algorithm—DBSCAN. The technologies identified are used in some cases in combination with technologies like computer vision, and Unmanned Aerial Vehicles (UAVs) from Industry 4.0 as well, and technologies traditionally used in the inland waterway and maritime transport sectors like AIS, GIS, and RADAR. This is not something that happens only in inland waterway transport for Industry 4.0 technologies. The same effect can be seen in other fields such as the Smart Manufacturing Systems (SMS) where the integration of these Industry 4.0 technologies can be applied to the creation of semi-autonomous industrial systems [106,107].

In emission reduction, the use of Industry 4.0 technologies is not very evident, and solutions in this domain are based on hard technologies such as engines with combined energy sources: LNG (Liquefied Natural Gas) and electricity and the use of alternative fuels.

In future works, a broader approach should be made, which collects articles from more scientific databases. It is suggested that future systematic literature reviews will be done in each of the domains detected in this work, in order to have a more detailed analysis of each strategy used to solve problems in inland waterway transport.

The main objective of this work was not to make a detailed analysis of each one of the technologies used in the solution of inland waterway transport problems. We consider that this work constitutes a first step in carrying out more in-depth reviews, which allow to understand possible advantages and disadvantages of Industry 4.0 technologies used in solving river transport problems. Therefore, future works should consider the environmental envelope: more sustainable systems, mitigation of environmental impacts, resilience of systems in the face of climate change, among others; construction systems: infrastructure, ports and storage systems, projects, among others; and project management: devices and systems for project monitoring, life cycle, among others.

## Figures and Tables

**Table 1:** Keywords used for the search queries.

Group	Keywords
Group 1	River navigation *, river transport *, inland waterway transport *
Group 2	Technology, logistic technology, river monitoring, navigation sensors, unmanned aerial vehicle, laser scanning, remote sensing.
Group 3	Artificial intelligence, machine learning, deep learning, big data, internet of things, Industry 4.0

* Any word that begins with the root/stem of the word truncated by the asterisk.

**Table 2:** Information sources used for the search phase.

Data Source	Type	URL
Scopus	Digital Library	http://www.scopus.com/ (accessed on 2 October 2021)
Web of Science	Digital Library	https://clarivate.com/webofsciencegroup/solutions/web-of-science (accessed on 2 October 2021)

**Table 3:** Search query algorithms.

Digital Library	Group	Algorithm
Scopus and Web of Science	Group 1 and group 2	TITLE-ABS-KEY ((“river navigation *” OR “river transport *” OR “inland waterway transport *”) AND (technology OR “logistic technology” OR “river monitoring” OR “navigation sensors” OR “unmanned aerial vehicle” OR “laser scanning” OR “remote sensing”)) AND PUBYEAR > 2016
Scopus and Web of Science	Group 1 and group 3	TITLE-KEY ((“river navigation *” OR “river transport *” OR “inland waterway transport*”) AND (“artificial intelligence” OR “machine learning” OR “deep learning” OR “big data” OR “Internet of things” OR “Industry 4.0”)) AND PUBYEAR > 2016

* Any word that begins with the root/stem of the word truncated by the asterisk.

**Table 4:** Clustering of the selected studies by domain.

Domain	Papers Selected
Traffic monitoring	[13,14,15,16,17,18,19,20,21,22,23,24,25,26,27,28,29,30,31,32,33,34,35,36,37,38,39,40,41,42,43,44,45,46,47]
Smart navigation	[48,49,50,51,52,53,54,55,56,57,58,59,60,61,62,63,64,65,66,67,68,69,70,71,72]
Emission reduction	[73,74,75,76,77,78,79,80,81,82,83,84,85,86,87,88]
Analytics with big data	[89,90,91,92,93,94,95]
Cybersecurity	[96,97,98,99,100]

**Table 5:** Strategies detected in papers reviewed to deal with traffic monitoring problems.

Strategy	Technologies	References
Trajectory prediction based on geopositioning data	- AIS, ECDIS, IoT - Genetic algorithms - Neural networks, Autoencoder - Long short-term memory (LSTM) - Recurrent neural networks (RNNs) - Kinematic interpolation - Density-based spatial clustering of -applications with noise (DBSCAN) - Bidirectional Gated Recurrent Unit (Bi-GRU) - GIS (Geographic Information System)	[13,14,15,16,18,21,22,24,25,26,29,30,33,35,36,42,89]
Images, video, and artificial vision methods for vessel detection	- Neural network Yolov3 - Neural network DarkNet53 - Otsu algorithm - SSD (Single Shot Multibox Detector - Ship detection dataset HRSC2016 - Ship dataset SSD2020, Google Earth - Global Navigation Satellite Systems Reflectometry (GNSS-R) - Remote sensing technology	[19,20,27,34,40,41,47]

**Table 6:** Strategies detected in papers reviewed to face the problems of smart navigation.

Strategy	Technologies	References
Automatic navigation and risk detection in real time	- Time Discrete Non-linear Velocity Obstacle (TD-NLVO) method. - AIS, RADAR. - Ship Heading Control Based on Fuzzy PID Control. - Deep reinforcement learning (DRL).	[52,53,54,56,62,71]
Collision risk detection from ports	- Deep learning - AIS. - 3D laser scanner	[49,51,66,67]

**Table 7:** Strategies detected in the papers reviewed to face the problems related to emission reduction.

Strategy	Technologies	References
Efficient ships and alternative fuels and the use of waste heat to generate electricity	- Steering system for push barges on the river - Engines with combined energy sources: LNG (Liquefied Natural Gas) and electricity - Thermoelectric generation modules	[75,78,80,87,88]
Ships propelled only with electric and solar energy	- Electric engines - Propellers for electric engines - Generation in ports with different techniques: wind, solar, hydro	[73,74,77,79]

## Data Availability

Not applicable.
